# Analysis of Cry1Ah Toxin-Binding Reliability to Midgut Membrane Proteins of the Asian Corn Borer

**DOI:** 10.3390/toxins12060418

**Published:** 2020-06-24

**Authors:** Sivaprasath Prabu, Muhammad Zeeshan Shabbir, Zhenying Wang, Kanglai He

**Affiliations:** State Key Laboratory for Biology of Plant Diseases and Insect Pests, Institute of Plant Protection, Chinese Academy of Agricultural Sciences, Beijing 100193, China; sivaprasathibt@gmail.com (S.P.); zeeshan@gdppri.com (M.Z.S.); zywang@ippcaas.cn (Z.W.)

**Keywords:** Cry1Ah toxin-binding proteins, Asian corn borer, pull-down assay, prophenoloxidase

## Abstract

Evolution of insect resistance to Bt toxins challenges the use of Cry toxins to control agricultural pests. In lepidopterans, Cry toxin affinity towards multiple midgut epithelial receptors has become a matter of dispute. Cry1Ah toxin-binding proteins were identified in the larval midgut of susceptible (ACB-BtS) and resistant (ACB-AhR) strains of the Asian corn borer (ACB). A pull-down assay was performed using biotinylated Cry1Ah toxin, and the binding proteins were identified by employing liquid chromatography–tandem mass spectrometry (LC-MS/MS). This study aimed to find the binding consistency of the midgut epithelial protein to the Cry1Ah toxin. The binding proteins from different fractions of SDS-PAGE showed a different pattern. We observed an isoform of prophenoloxidase PPO1b (UniProt Acc No. A0A1Q1MKI0), which was found only in the ACB-AhR fractions. Prophenoloxidase (proPO) is an extraordinary defense molecule activated in insect species during pathogen invasion and the wound healing process. Importantly, this prophenoloxidase might have direct/indirect interaction with the Cry1Ah toxin. Our data also suggest that factors like techniques, enrichment of binding proteins in the sample and the reversible and irreversible nature of the brush border membrane vesicles (BBMVs) to Cry toxins could cause the inconsistency in the protein–protein interactions. Moreover, inside the larva midgut, the influence of the Cry toxins under physiological conditions might be different from the laboratory procedures.

## 1. Introduction

The *Bacillus thuringiensis* (Bt) insecticidal crystal (Cry) proteins are a diverse family of proteins with over 780 identified members [1]. Bt Cry proteins have insecticidal properties. The wide variety of these toxins, their effectiveness and relatively inexpensive processing have made Bt the world’s most commonly used biopesticide. These Cry proteins are used as sprays or as a Cry gene expressing transgenic crops, and are used mainly in the fight against lepidopteran and other agricultural crop pests [2]. However, the extensive use of transgenic crops raises the selection pressure to evolve insect resistance to the Bt proteins, thus decreasing the effectiveness of the Bt Cry proteins [3,4,5]. Several cases of insect resistance to Bt toxins both in the field and under laboratory conditions have been reported [4,5,6,7,8].

Hence, understanding the mechanism of insect resistance to Cry toxins is essential for the development of effective pest management strategies. Previously, a study showed that the reduction in toxin-binding to the midgut epithelium is associated with the resistance evolution mechanism in different insect populations [9]. Previously, two different models explained the modes of action delivered by the Cry toxin: (1) the pore formation model and (2) the signal transduction model [10]. The pro-toxins are cleaved by midgut proteases that bind to the membrane proteins in the brush border membrane vesicles (BBMVs) of the insect midgut. Eventually, these toxic oligomers promote insertion or activation of protein kinase A, which primes to osmotic imbalance or cytotoxicity and ultimately leading to cell death [11,12,13,14,15]. Several approaches have been shown to be successful in dealing with insect resistance, such as the acquisition of novel Cry proteins with different modes of action [6].

In Lepidoptera, different proteins have been previously identified as Cry1A toxin receptors, like cadherin (CAD) [12,16,17,18], aminopeptidase-N (APN) [19,20,21] and alkaline phosphatase (ALP) [22,23,24]. In addition, other different proteins have been reported, such as the ATP-binding cassette (ABC—ABCC2, ABCC3 and ABCA2) transporter [25,26,27,28], actin, Hsp70 and V-ATPase [29,30,31]. In addition to all these membrane proteins, it was reported that immune-related proteins might also contribute to the development of resistance [32,33,34,35,36]. Recently, a study brought evidence of the possible direct binding of the Cry1Ah toxin to phenoloxidase (PO) and other immune-related proteins identified in BBMV samples extracted from susceptible and resistant strains of ACB [36]. However, additional proteins may still be involved in the interaction with the Cry1A toxin.

The Asian corn borer (ACB), *Ostrinia furnacalis* (Guenée) (Lepidoptera: Crambidae), is an important lepidopteran pest, with larval caterpillars causing the most destructive damage to maize throughout China [37,38,39]. The larvae infest most parts of the plant, but the ears and stalk are the primary victims [40]. Managing this pest is a difficult task for the farmers due to the cost of insecticides, resistance development, environmental concerns and uncertainty about the effectiveness of the pest management strategies [41]. Shabbir et al. [39] proved that the Cry1Ah-expressing maize is effective against ACB larvae. However, in the same study, the ACB larvae gradually developed resistance against the Cry1Ah toxin (200-fold) as well as various levels of cross-resistance against the Cry1Ab, Cry1Ac and Cry1Fa toxins under laboratory conditions. The exact mechanism for the development of resistance in the ACB remains unknown.

In this study, we have determined the consistency of the Cry1Ah toxin-binding to BBMV proteins extracted from susceptible (ACB-BtS) and resistant (ACB-AhR) strains of the ACB. We employed a different protocol to analyze the consistency of the Cry1Ah toxin-binding protein and compared the pattern of the ACB proteins interacting with the Cry1Ah toxin. The Cry1Ah affinity towards the BBMV proteins was analyzed via a pull-down assay and LC-MS/MS analysis.

## 2. Results

### 2.1. APN and ALP Activities in ACB Larval Midgut

The APN and ALP activities in the BBMV samples were approximately three- to four-fold higher than in the midgut homogenate (Table 1). Enrichment of BBMV proteins was indicated by the high ratios of APN and ALP activities in the BBMV sample extracted from the midgut homogenate. 

### 2.2. Cry1Ah-Binding Proteins from BBMV of ACB

**Figure 1:**
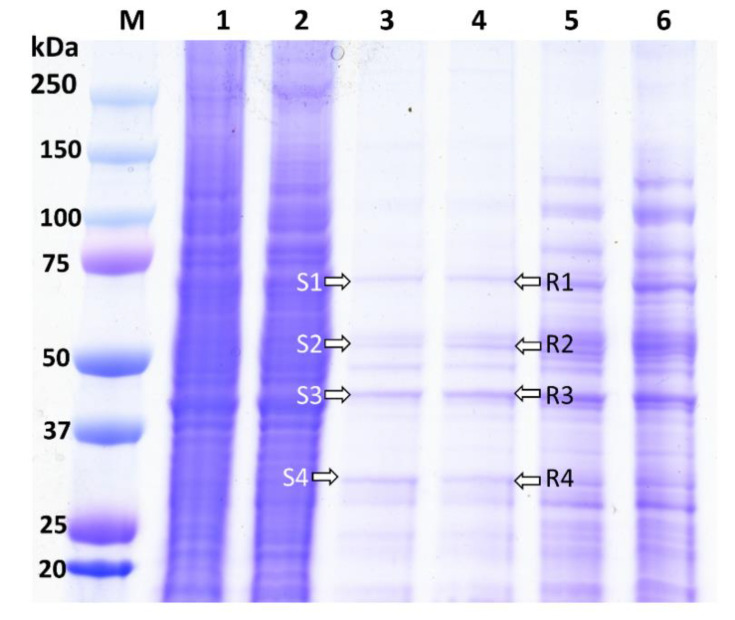
Pull-down Cry1Ah-binding proteins from the BBMVs of the ACB-BtS and ACB-AhR strains. M: Precession plus, dual color marker (Biorad, Hercules, CA, USA); Lane 1 and 2: BBMV from ACB-BtS and ACB-AhR strains; Lane 3 and 4: Cry1Ah-binding proteins finally eluted from the BBMVs of the ACB-BtS and ACB-AhR strains; Lane 5 and 6: Non-treated control proteins from the ACB-BtS and ACB-AhR strains. S1–S4 and R1–R4, bands selected for LC-MS/MS analysis.

The biotinylated Cry1Ah interacted with the BBMV proteins extracted from the ACB-BtS and ACB-AhR strains and were analyzed using 12% Gel (Figure 1). The ACB proteins that are bound to Cry1Ah are shown from Lanes 3 and 4 (Figure 1). Lanes 1 and 2 show the extracted BBMV proteins of the ACB-BtS (7.85 mg/mL) and ACB-AhR (9.20 mg/mL) strains, respectively. Biotinylated Cry1Ah interacted with the BBMVs of both the ACB-BtS and ACB-AhR strains, and showed a similar binding pattern in Lanes 3 and 4 (Figure 1). 

### 2.3. Cry1Ah-Binding Proteins Identified from the BBMVs of the ACB

The Cry1Ah-binding proteins of the ACB from Lanes 3 and 4 were divided into four fractions (Figure 1). To identify the Cry1Ah-binding proteins, these fragments of the gel were subjected to LC-MS/MS analysis. The proteins identified in each fraction of the gel showed a positive hit with a higher score, more than the threshold (>0.05) level (Table 2). The corresponding accession numbers obtained from the UniProt database are shown in Table 2. LC-MS/MS results revealed 32 different proteins binding Cry1Ah in the BBMV from ACB-BtS and 33 proteins from ACB-AhR (Table 2). Among these proteins, 24 Cry1Ah-binding proteins were common in both populations (Table 2). Besides the commonly reported Cry-binding proteins, such as APN, ALP, cadherin, Hsp70 and V-ATPase, we also detected prophenoloxidase, prophenoloxidase PPO1a, serine proteinase inhibitor 2 and serpin 5 from the S3, R3 and S4 fractions (Table 2). Especially, there were six proteins, namely carboxylesterase, ryanodine receptor, beta-hexosaminidase, NADH-ubiquinone oxidoreductase, acyl-CoA delta-9 desaturase and ALP, found only in the ACB-BtS (Table 2). On the other hand, integrin beta, prophenoloxidase and trehalase were detected only in the ACB-AhR samples (Table 2, Supplementary Table S1). ALP was identified only in the ACB-BtS samples, and thus not detected in the ACB-AhR samples (Supplementary Table S1). 

## 3. Discussion

In the present study, we analyzed the consistency of Cry1Ah toxin-binding to specific BBMV proteins extracted from the susceptible ACB-BtS and resistant ACB-AhR strains of *O. furnacalis.* The list of Cry1Ah-binding proteins obtained from the four fractions (Figure 1) with their respective molecular weight was compared with the Cry1Ah-binding proteins previously reported by Shabbir et al. [36]. The Cry toxins that bind to the BBMV protein receptors are very important for understanding the toxic nature of the Cry toxins [42]. Interaction between the Bt toxins and BBMVs is associated with the evolution of resistance mechanisms [43,44,45,46,47]. 

In this study, we found some proteins that were previously reported as Cry-binding proteins, namely APN, ALP, cadherin, Hsp70 and V-ATPase [29,30,31,48] (Table 2). We compared the list of Cry1Ah-binding proteins of the ACB-BtS and ACB-AhR strains. In total, six proteins, namely carboxylesterase, ryanodine receptor, beta-hexosaminidase, NADH-ubiquinone oxidoreductase, acyl-CoA delta-9 desaturase and ALP, were only detected in the ACB-BtS samples (Table 2). Integrin beta, prophenoloxidase and trehalase were only detected in the ACB-AhR fractions (Table 2). Although most of the identified proteins seem to be similar to the binding protein reported [36], we observed few proteins which were different from that list. Likewise, in our present study, prophenoloxidase PPO1b (UniProt ID: A0A1Q1MKI0) and trehalase (UniProt ID-A0A1B2AQF4) were found only in the ACB-AhR fractions (Table 2, Supplementary Table S1). This inconsistency in binding might be associated with receptors on the BBMVs, including both reversible and irreversible binding steps [49,50].

On the other hand, we selected four fractions from the SDS-PAGE that had similar molecular weights as reported by Shabbir et al. [36] (Figure 1) and compared with the list of proteins from the fractions S3, S4, S6, S7–R3, R4, R6 and R7. Firstly, our protein list from the S1 and R1 fractions was compared with S3 and R3. Shabbir et al. [36] reported 14 proteins from the ACB-BtS samples and 11 from the ACB-AhR samples; however, we found 18 binding proteins from the ACB-BtS samples and 16 from the ACB-AhR samples. Arginine kinase, APN, actin, Hsp 7090, prophenoloxidase PPO1a and V-ATPase were the common proteins identified from both studies. Besides, proteins like trypsin, juvenile hormone and prophenoloxidase PPO1b were not found in our fractions. Inconsistency in binding may be related to the experimental setup or chemicals that were used for the study. Shabbir et al. [36] used the NHS-activated sepharose (GE Healthcare, Uppsala, Sweden), which was different from our pull-down assay protocol. An earlier report, based on a heterologous competition binding assay, proposed that Cry1Ab and Cry1Ac share the same binding sites in *L. dispar* [51], but the results suggested by Wolfersberger are contradictory to the ligand blotting results [52]. In *Manduca sexta*, partially purified Cry1Ac binding APN has been used in competition binding assays, surface plasmon resonance experiments and liposome reconstitution experiments [53,54,55]. Competition binding assays and liposome reconstitution experiments showed the affinity of Cry1Ac to APN was relatively low. Conversely, surface plasmon resonance results differed between the reports. This matter created a huge confusion among the researchers to adapt the right experiment to find the Cry toxin-binding protein. Such differences can also correlate in these experiments with the enrichment of binding proteins at different degrees that are subject to the Cry1A toxins [56].

Comparing the other fractions (S2 to S4), the pattern of the binding protein seems to differ from Shabbir et al. [36] (Table 2, Supplementary Table S2) Several factors might affect the interaction between the Cry1Ah toxin and binding protein, like using different kit chemicals, enrichment of binding proteins after the extraction process and the reversible and irreversible nature of the BBMVs to Cry toxins [49,50,56]. Moreover, inside the larva midgut, the influence of the Cry toxins under physiological conditions might be different from the experiments carried out in the laboratory. At this point, understanding the mechanism of the midgut receptors towards Cry toxins will be a vital part to maintain the right toxin under field conditions. However, based on these results, we cannot conclude the exact list of binding proteins from the BBMVs. Remarkably, in accordance with Shabbir et al. [36], ALP was detected only in the BBMV samples of the ACB-BtS strain, suggesting that reduced levels of ALP expression may contribute to the development of resistance in the ACB-AhR strain. *Heliothis virescens* and *H. armigera* showed resistance to Cry1Ac, and *Spodoptera frugiperda* developed resistance against the Cry1Fa toxin, suggesting that reduced levels of ALP protein promote the resistance development in these lepidopteran pests [57,58].

However, the expression of the ALP gene was 6-fold higher in ACB-AhR than in ACB-BtS. Similarly, ALP enzymatic activity in the BBMV samples of the ACB-AhR strain was two-fold higher than for the ACB-BtS strain [36]. Interestingly, we did not find any ALP isoforms in the pull-down samples from ACB-AhR. Based on our pull-down results, we suspect a possible mutation might occur in the ALP gene. In *H. virescens*, a mutation in the midgut cadherin protein inactivated the 12-cadherin domain and also showed resistance to Cry1A toxins [59]. Similarly, combined mutation in cadherin and ABCC2 (ATP Binding Cassette Subfamily C Member 2) proteins in *H. virescens* promoted a high level of resistance to Cry1Ac and eliminated the binding affinity to Cry1Aa, Cry1Ab and Cry1Ac [59]. Gene mapping and sequence techniques revealed a mutation in the homologue of ABCC2 in *Plutella xylostella* [60]. Vice versa, a Cry1Ab-β16-L511A mutation affected the binding of the Cry1Ab toxin to ALP in *M. sexta* larvae [23]. The occurrence of single-site mutations in the conserved arginine region of CryIAa affect the formation of the ionic channel [61]. Altogether, the transgenic method delivers convincing evidence that even a small structural change in the Cry toxin-binding protein can have a great impact on Cry toxicity [62]. These facts highlight the importance of knowledge on mutagenesis and its possible contribution for disabling the protein–protein interactions and enhance the development of resistance in the target pests. 

In a different view, we observed certain immune-related proteins were in the binding protein list. We detected prophenoloxidase, prophenoloxidase PPO1a, serine proteinase inhibitor 2 and serpin 5 from the different fractions. The effectiveness of certain bioinsecticides may be correlated to the activation of proPO [63]. PO levels of *Helicoverpa armigera* and *S. frugiperda* were elevated after the larvae were exposed to Cry1Ac toxin [32,34]. In the event of Cry toxicity, the midgut epithelium was gradually breached by the Cry toxins. Activated phenoloxidase (PO) was regulated by the isoforms of the serine proteases and activated the formation of quinones, and these quinones are reactive intermediates for repairing tissue damage [64,65,66]. Our results show a possibility that the Cry1Ah toxin may interact with proPO and other regulating proteins like serine proteinase inhibitor 2 and serpin 5 that were identified in samples from both susceptible and resistant strains of the ACB. A further in-depth study would render the importance of these immune-related proteins and its action towards Cry toxicity. However, we cannot conclude that these proPO molecules possess an affinity to bind directly to the Cry1Ah toxin. Intensive studies are needed to confirm the direct interactions. Protein expression, the yeast two-hybrid system and other techniques could be helpful to determine the direct interaction of the proPO/related proteins with the Cry1Ah toxin. 

Overall, our results suggest the presence of a binding inconsistency between the BBMV binding proteins and Cry1Ah toxin after the pull-down assay. There might be several factors influencing the binding ability, these factors including techniques, enrichment of binding proteins in the sample and the reversible and irreversible nature of the BBMVs to Cry toxins. These factors may play a major role in protein–protein interactions. Furthermore, another important point must be accounted for during Cry toxicity in insects: After the model pest is subjected to the Cry toxin, the actual events take place under physiological conditions, which might differ from the experiments that we employed to understand the Cry toxin interactions with the BBMVs. This work would provide good interest to the readers; for example, readers can compare the patterns of the gel pictures from this work and from that of Shabbir et al. [36] as well. The patterns and lists of binding proteins would render them a new idea regarding these Cry toxin-binding proteins. On the other hand, ALP was not detected in the ACB-AhR samples; this finding might provide interest to further research the ALP mutation in the ACB-AhR strain. At this point, a precise method is needed to assess the Cry toxin mode of action. A pull-down assay alone cannot conclude the binding mechanism of the Cry toxin, and there are multiple proteins reported for their affinity towards the Cry toxin. Among them, tracking a high-affinity protein to a particular Cry toxin would enable a novel way to control or delay the resistance development in a given crop pest. To achieve this, engaging the purified binding proteins with Cry toxins by employing multiple conformational experiments would render a clear understanding on Cry toxicity.

## 4. Conclusions

Taken together, up-to-date the Cry toxin mode of action remains unclear. We investigated and observed the inconsistency in the Cry1Ah toxin-binding proteins from the BBMVs of the ACB. There remain important factors that might influence the binding specificity of midgut BBMV proteins towards Cry toxins. These inconsistencies may have arisen due to several factors influencing their binding ability, and these factors include techniques employed to find protein–protein interactions, enrichment of binding proteins in the sample and the reversible and irreversible nature of the BBMVs to Cry toxins. Understanding Cry toxicity will remain difficult, until a crucial or precise method is introduced to demonstrate the correct receptor(s) interacting with the Cry toxin.

## 5. Materials and Methods

### 5.1. Insect Strains

Susceptible (ACB-BtS) and Cry1Ah (ACB-AhR) resistant strains of *O. furnacalis* used in this study were obtained from the Institute of Plant Protection (IPP), Chinese Academy of Agricultural Sciences (CAAS), Beijing, and reared under laboratory conditions. Larvae were reared at 27 ± 1 °C, 70–80% relative humidity (RH), with a photoperiod of 16:8 h light:dark (L:D). Fourth instar second-day larvae were used in this study. Purified Cry1Ah (trypsin-activated) toxin was purchased from Beijing General pest Biotech Research Co., Ltd., Beijing, China. The Cry1Ah toxin was expressed in *B. thuringiensis*, a crystalliferous mutant (HD73-).

### 5.2. Preparation of BBMV

Midgut tissue was dissected from the ACB-BtS and ACB-AhR strains. About 200 larvae were used for BBMV extraction for each strain. The midgut tissue was excised from the foregut and hindgut, and a longitudinal slit was made in the midgut tissue to remove the food and other debris. Dissected midgut tissue was rinsed with ice-cold Mannitol buffer (300 mM Mannitol, 17 mM Tris-HCl, 5 mM EGTA, 2 mM DDT and 0.5 mM PMSF, pH 7.4) and the tissue was picked with fine forceps and put on filter paper to remove the excess liquid. The BBMV was prepared according to the Wolfersberger method [67]. Briefly, 200 mg of midgut tissue sample was weighed and transferred into the Dounce homogenizer (7 mL), and a five-fold volume (*w*/*v*) of Mannitol buffer was added and homogenized on ice for five minutes and allowed for incubation on ice for two minutes; this process was repeated three times. The homogenate was diluted with an equal volume of MgCl_2_ (24 mM, pH 7.6) and incubated on ice for 15 min. Initially, this mixture was centrifuged at 4500 rpm for 15 min at 4 °C to remove the cell debris; the pellet was discarded, and the supernatant was retained. Further, the supernatant was centrifuged at 16,000 rpm for 30 min at 4 °C, and the pellet corresponds to the BBMV. The resulting pellet was suspended in 0.5 mL Mannitol buffer, and an equal volume MgCl_2_ (24 mM, pH 7.6) was added. This sample was centrifuged under the same conditions mentioned above and this process was repeated twice. The pellet (BBMV) was solubilized using 1 mL of solubilization buffer (Octyl β-d-glucopyranoside 1%, 50 mM Na_2_HPO_4_/NaH_2_PO_4_, 5 mM EGTA, 50 mM NaCl, 5 mM EDTA, 0.5 mM PMSF, pH 7.5) and incubated at 4 °C for 1 h and centrifuged at 50,000 rpm for 30 min. The supernatant was recovered, and the protein concentration in the BBMV was determined by the Bradford method using BSA as standard [68]. To determine the purity of BBMV, the APN and ALP enzymatic activities were assessed in the midgut tissue homogenate and the BBMV samples extracted from the ACB-BtS and ACB-AhR strains [69].

### 5.3. Pull-Down Assay

The purified Cry1Ah toxin was biotinylated using EZ-Link NHS-LC-Biotin (Thermofisher, Waltham, MA, USA). NHS-LC-Biotin was dissolved in 300 µL of N,N-Dimethylformamide (DMF) and then added to 4.7 mL of activated Cry1Ah toxin (2 mg/mL) in sodium carbonate buffer (50 mM, pH 10). The mixture was incubated for overnight at 4 °C and dialyzed (Biotopped tubing, 15 kDa) in sodium carbonate buffer (50 mM, pH 10) for 6 h at 4 °C. The Pierce™ Pull-Down Biotinylated Protein: Protein Interaction Kit (Thermoscientific, Waltham, MA, USA) was used to perform the pull-down assay following the manufacture’s protocol. To a spin column, 100 μL of biotinylated bait protein (NHS-LC-Biotin coupled with Cry1Ah) and 50 μL of streptavidin gel slurry was added and incubated at 4 °C for 45 min and centrifuged (1250× *g*, 4 °C, 1 min). After the incubation, 250 μL of biotin blocking solution was added to the column and incubated at RT for 5 min and centrifuged (1250× *g*, 4 °C, 1 min); this step is to block the available streptavidin sites. A total of 100 μL of BBMV (prey protein) was added and incubated for 2 h at 4 °C and centrifuged (1250× *g*, 4 °C, 1 min). Following the incubation, 250 μL of wash buffer was added to the column and mix by gently inverting the columns 5–7 times and centrifuged (1250× *g*, 4 °C, 1 min). A total of 10 μL of the neutralization buffer was added to the column, which will neutralize the pH of the contents upon elution. Finally, the Cry1Ah-binding proteins were eluted by adding 250 μL of elution buffer into the column and incubated at RT for 5 min and centrifuged (1250× *g*, 4 °C, 1 min). A non-treated gel control (without bait protein) was maintained. The columns were sealed with bottom plugs during the incubation or adding of buffers. Three technical replications were maintained per sample.

### 5.4. Identification of Cry1Ah-Binding Proteins

These eluted proteins were analyzed in a 12% Express Plus TM SDS-PAGE (Genscript, Piscataway, NJ, USA). The gel was stained using EZ blue™ Gel staining reagent (Sigma, St. Louis, MO, USA). The binding proteins of the ACB-BtS and ACB-AhR strains from Lanes 3 and 4 with different molecular weights were selected and cut into four fractions. Three technical replications were maintained per sample. All the fractions were sent to the Beijing Bio-Tech Pack Technology Company Ltd. (Beijing, China). for protein identification by liquid chromatography–tandem mass spectrometry (LC-MS/MS). The following parameters were set during the LC-MS/MS analysis: the protein modifications were carbamidomethylation (C) (fixed), oxidation (M) (variable), enzyme specificity was set to trypsin, the maximum missed cleavages were set to 2, the precursor ion mass tolerance was set to 10 ppm and the MS/MS tolerance was set to 0.6 Da. The peptides identified with high confidence were chosen for the downstream protein identification analysis. The raw mass spectrometry files were analyzed using Maxquant (1.5.6.5) and matched against the protein databases. Identifications of proteins were accepted if they could be established at a probability of >95% and contained at least two of the peptides identified. 

## Figures and Tables

**Table 1:** Enzymatic activities of aminopeptidase-N (APN) and alkaline phosphatase (ALP) in the midgut homogenate and brush border membrane vesicles (BBMVs) from resistant (ACB-AhR) and susceptible (ACB-BtS) Asian corn borer (ACB) larvae.

Enzymatic Assays (μmol/min/mL)	ACB-BtS		ACB-AhR	
	Midgut Homogenate	BBMV	Midgut Homogenate	BBMV
APN	16.92	52.40	26.13	98.45
ALP	9.42	32.42	16.68	62.15

**Table 2:** The LC-MS/MS analysis results of the Cry1Ah-binding proteins of the BBMVs from susceptible and resistant strains of *Ostrinia furnacalis*.

ACB-BtS Midgut Fractions					ACB-AhR Midgut Fractions				
Fraction	Score	Accession Number	Protein Description	Sequence Coverage %	Fraction	Score	Accession Number	Protein Description	Sequence Coverage %
S1	65.30	tr|B5A8 × 9	Aminopeptidase	7.2	R1	22.56	tr|A0A0A7BYG4	Elongation factor 1 alpha	9.6
	20.09	tr|D2Y440	Actin	19.9		19.68	tr|E5LEV4	Heat shock 70	4.7
	17.09	tr|Q8WB29	Cytochrome c oxidase subunit 3	2.7		15.00	tr|A0A0F7QEA3	Actin	20.4
	16.87	tr|A0A0A7BYG4	Elongation factor 1 alpha (Fragment)	7.6		21.40	tr|B5A8X9	Aminopeptidase	1.2
	15.68	tr|E5LEV4	Heat shock 70 kDa	3.0		6.05	tr|A0A1B4ZBI6	Odorant binding protein	5.9
	14.34	tr|B5A8Y0	Aminopeptidase	4.1		3.26	tr|Q2PQR1	Ubiquitin	10.6
	12.45	tr|Q8W7D1	ATP synthase subunit a	3.4		2.90	tr|A0A0A7DWF3	Arginine kinase	2.0
	6.48	tr|E3W6T9	V-ATPase subunit A	3.9		2.44	tr|A0A0E4B3V9	Odorant receptor	4.2
	5.28	tr|A0A0A7BYG7	Glyceraldehyde-3-phosphate dehydrogenase	8.6		59.08	tr|A7LIA2	Cadherin-like protein	3.7
	5.10	tr|A0A0F7QEE5	Carboxylic ester hydrolase	1.3		11.56	tr|A0A0B6VK98	Uncharacterized protein	2.0
	4.66	tr|Q8WB23	Cytochrome	2.4		6.99	tr|A0A0F7QIG2	Ionotropic receptor	1.1
	3.97	tr|A0A0F7QIG2	Ionotropic receptor	2.4		1.57	tr|A0A1Q1MKI0	Prophenoloxidase PPO1b	4.2
	3.84	tr|Q2PQR1	Ubiquitin	24.3		13.74	tr|A0A0A7BYG4	Elongation factor 1 alpha	14.7
	3.69	tr|A0A1B4ZBI6	Odorant binding protein	6.0		12.40	tr|Q8W7D1	synthase subunit a	3.1
	2.95	tr|A0A0A7BYS7	Glutathione-S transferase (Fragment)	4.3		10.17	tr|Q8WB24	NADH dehydrogenase subunit 6	5.6
	2.90	tr|A0A0A7DWF3	Arginine kinase	2.0		9.14	tr|Q8WB23	Cytochrome b	9.4
	2.34	tr|A0A0A7BYM5	Glutathione-S transferase	6.0		8.85	tr|Q2V6H3	Chitin synthase	0.5
	2.13	tr|E0XJK4	Heat shock protein 90	1.0		8.45	tr|A0A0F7QJX1	Carboxylic ester hydrolase	2.0
	7.59	tr|A0A1Q1MKI5	Prophenoloxidase PPO1a	3.1					
	1.65	tr|A0A0B6VK98	Uncharacterized protein	2.0					
	1.44	tr|B4YIR0	Chitin synthase	2.2					
S2	240.08	tr|D0UYB1	Aminopeptidase	44.2	R2	64.86	tr|B5A8X9	Aminopeptidase	15.0
	181.29	tr|A7YAH7	Aminopeptidase	27.4		45.15	tr|A0A0S2C6J0	Actin	14.1
	123.85	tr|B5A8Y0	Aminopeptidase	19.9		59.08	tr|A7LIA2	Cadherin-like protein	3.2
	49.61	tr|A7LIA2	Cadherin-like protein	7.6		28.02	tr|A0A0A7BYG4	Elongation factor 1 alpha	7.1
	29.97	tr|E3W6T9	V-ATPase subunit A	17.7		6.46	tr|B5A8X9	Aminopeptidase	1.5
	21.42	tr|Q7K403	Acyl-CoA delta-9 desaturase	5.1		17.85	tr|A0A0A7BYG7	Glyceraldehyde-3-phosphate dehydrogenase	4.5
	18.31	tr|A5JJU1	Aminopeptidase	23.9		14.97	tr|A7YAH7	Aminopeptidase	2.3
	14.21	tr|E5LEV4	Heat shock 70 kDa	2.8		13.21	tr|E5LEV4	Heat shock 70 kDa	1.7
	14.18	tr|A0A0S2C6J0	Actin	18.5		11.56	tr|A0A0B6VK98	Uncharacterized protein	2.0
	6.24	tr|Q8WB29	Cytochrome c oxidase subunit	5.3		7.31	tr|A0A0A7DWF3	Arginine kinase	2.0
	5.90	tr|D0UYB2	Aminopeptidase	40.2		6.72	tr|Q2PQR1	Ubiquitin	11.8
	5.18	tr|A0A0F7QEE5	Carboxylic ester hydrolase	1.3		5.95	tr|L7QRW6	Period	2.2
	2.88	tr|M4Q143	NADH-ubiquinone oxidoreductase	3.1		5.87	tr|A0A1B2AQF4	Trehalase	1.1
	1.70	tr|Q2PQR1	Ubiquitin	28.9		5.77	tr|E3W6T9	V-ATPase subunit A	1.5
	1.68	tr|A0A0A7BYG7	Glyceraldehyde-3-phosphate dehydrogenase	2.1		5.73	tr|A0A0F7QEC7	Aldehyde oxidase	1.4
	1.60	tr|A0A1B4ZBI6	Odorant binding protein 9	5.9		5.71	tr|A0A1Q1MKI5	Prophenoloxidase PPO1a	3.6
	1.30	tr|M1RM07	Elongation factor-1 alpha	14.1		5.68	tr|A0A0E4B3V9	Odorant receptor	2.0
	1.04	tr|Q8WB31	Cytochrome c oxidase subunit 1	1.6					
	0.95	tr|A0A0E4B5I4	Odorant receptor	1.7					
	0.57	tr|A0A0A7BYS7	Glutathione-S transferase	3.2					
	0.49	tr|L7QRW6	Period	2.2					
	0.49	tr|A0A0E3VLQ5	Odorant receptor	2.3					
	0.45	tr|M1RTC5	Elongation factor-1 alpha	16.7					
S3	323.31	tr|E3W6T9	V-ATPase subunit A	71.5	R3	244.29	tr|B5A8X9	Aminopeptidase	28.9
	95.44	tr|B5A8X9	Aminopeptidase	21.7		66.82	tr|A7YAH7	Aminopeptidase	9.2
	50.77	tr|E5LEV4	Heat shock 70 kDa	28.2		65.03	tr|E3W6T9	V-ATPase subunit A	14.3
	37.45	tr|A7YAH7	Aminopeptidase	12.2		54.05	tr|B5A8Y0	Aminopeptidase	10.6
	30.53	tr|E0XJK4	Heat shock protein 90	17.3		27.18	tr|D2Y440	Actin	30.6
	17.39	tr|B5A8Y0	Aminopeptidase	8.0		25.37	tr|D0UYB2	Aminopeptidase	7.7
	14.82	tr|A0A0A7BYG4	Elongation factor 1 alpha	14.7		21.40	tr|B2LS41	Aminopeptidase	26.9
	9.53	tr|A0A1L7B974	Serpin 5	12.2		21.13	tr|A0A0S2C6J0	Actin	23.9
	12.66	tr|A0A0F7QJX1	Carboxylic ester hydrolase	9.6		14.45	tr|A0A0A7BYG4	Elongation factor 1 alpha	7.1
	9.12	tr|B2LS41	Aminopeptidase	21.7		13.98	tr|E5LEV4	Heat shock 70 kDa	2.8
	7.98	tr|D0UYB2	Aminopeptidase	5.9		13.91	tr|A0A0A0YWU7	Arginine kinase	5.6
	7.98	tr|D2Y440	Actin (Fragment)	19.1		6.62	tr|A0A1Q1MKI0	Prophenoloxidase PPO1b	1.7
	7.83	tr|A0A0A7DWF3	Arginine kinase	8.5		6.29	tr|A0A0F7QIG2	Ionotropic receptor	1.1
	7.72	tr|Q06GJ0	Beta-hexosaminidase	2.7		6.09	tr|Q8WB29	Cytochrome c oxidase subunit 3	2.7
	4.99	tr|M1RTC5	Elongation factor-1 alpha	22.3		5.71	tr|J7FBQ4	Odorant receptor	2.4
	4.83	tr|A0A0S2C6J0	Actin	14.1		5.60	tr|M1RTC5	Elongation factor-1 alpha	5.6
	3.62	tr|M1RM07	Elongation factor-1 alpha	19.4		5.60	tr|A0A0F7QEJ4	Sensory neuron membrane protein 1	1.5
	2.96	tr|Q2PQR1	Ubiquitin	24.9		2.43	tr|A0A1L7B973	Serine proteinase inhibitor 2	17.5
	2.64	tr|Q8WB31	Cytochrome c oxidase subunit 1	1.6		14.59	tr|A0A1L7B974	Serpin 5	12.4
	1.84	tr|G1JT78	Alkaline phosphatase	1.3					
S4	258.25	tr|E3W6T9	V-ATPase subunit A	49.2	R4	231.15	tr|E3W6T9	V-ATPase subunit A	27.1
	49.45	tr|B5A8X9	Aminopeptidase	14.4		164.37	tr|B2LS41	Aminopeptidase	16.6
	24.74	tr|A7YAH7	Aminopeptidase	8.7		48.18	tr|D2Y440	Actin	30.2
	23.70	tr|A0A1L7B973	Serine proteinase inhibitor 2	14.6		46.51	tr|B5A8X9	Aminopeptidase	16.8
	19.10	tr|A0A0F7QJX1	Carboxylic ester hydrolase	13.3		43.97	tr|A0A0S2C6J0	Actin	28.3
	17.18	tr|A0A0F7QIF1	Carboxylic ester hydrolase	15.0		37.32	tr|E0XJK4	Heat shock protein 90	6.3
	10.56	tr|E0XJK4	Heat shock protein 90	7.8		30.01	tr|E5LEV4	Heat shock 70 kDa	6.3
	6.84	tr|A0A0S2C6J0	Actin	17.9		24.14	tr|A0A0A7BYG4	Elongation factor 1 alpha	7.1
	6.06	tr|A0A0B6VQ49	Uncharacterized protein	42.7		22.07	tr|F6MEP1	Storage protein	6.1
	5.24	tr|D2Y440	Actin	19.1		8.94	tr|Q8WB29	Cytochrome c oxidase subunit 3	2.7
	4.90	tr|A0A0A7BYG4	Elongation factor 1 alpha	11.2		8.02	tr|M1RTC5	Elongation factor-1 alpha	9.4
	4.58	tr|M4T4G3	Ryanodine receptor	1.3		7.72	tr|A0A0F7QEE5	Carboxylic ester hydrolase	1.3
	4.37	tr|A0A0A7DWF3	Arginine kinase	8.5		6.74	tr|A0A1B4ZBI6	Odorant binding protein 9	5.9
	4.23	tr|B5A8Y0	Aminopeptidase	2.2		6.65	tr|A0A0A7BYG7	Glyceraldehyde-3-phosphate dehydrogenase	2.1
	3.00	tr|M1RM07	Elongation factor-1 alpha	14.1		6.40	tr|A0A1Q1MKI5	Prophenoloxidase PPO1a	1.2
	1.81	tr|Q8WB29	Cytochrome c oxidase subunit 3	2.7		6.28	tr|A2TIK8	Integrin beta	1.1
	1.67	tr|E5LEV4	Heat shock 70 kDa	1.1		5.98	tr|A0A0A7BYS7	Glutathione-S transferase	3.2
	1.48	tr|Q2V6H3	Chitin synthase	3.0					
	1.33	tr|D2KWQ3	Odorant receptor	2.5					
	1.20	tr|G1JT78	Alkaline phosphatase	1.3					
	1.08	tr|B2LS41	Aminopeptidase	12.7					
	0.89	tr|A0A1L7B974	Serpin 5	22.9					
	0.87	tr|A0A1U8ZSW0	Storage protein	1.2					
	0.85	tr|A0A0F7QEC9	Carboxylesterase	1.4					

## Supplementary Materials

The following are available online at https://www.mdpi.com/2072-6651/12/6/418/s1, Table S1: List of different Cry1Ah binding protein BBMVs from susceptible and resistant strains of *Ostrinia furnacalis*, Table S2: List of common Cry1Ah binding protein of BBMVs from susceptible and resistant strains of *Ostrinia furnacalis*.
