# Supplementary material for: Analysis of Cry1Ah Toxin-Binding Reliability to Midgut Membrane Proteins of the Asian Corn Borer
Source: Toxins (Basel). 2020 Jun 24;12(6):418. doi: 10.3390/toxins12060418 (PMC7354594; doi:10.3390/toxins12060418)
Supplement: Supplementary file 1 [file toxins-12-00418-s001.pdf]

Supplementary Materials: Analysis of Cry1Ah Toxin-Binding Reliability to Midgut Membrane Proteins of the Asian Corn Borer

Sivaprasath Prabu, Muhammad Zeeshan Shabbir, Zhenying Wang and Kanglai He

Table S1. List of different Cry1Ah binding protein BBMVs from susceptible and resistant strains of *Ostrinia furnacalis*.

ACB-BtS Midgut Fractions					ACB-AhR Midgut Fractions				
**Fraction(s)**	**Score**	**Accession Number**	**Protein Description**	**Sequence Coverage %**	**Fraction(s)**	**Score**	**Accession Number**	**Protein Description**	**Sequence Coverage %**
S4	0.85	tr∣A0A0F7QEC9	Carboxylesterase	1.4	R4	6.28	tr∣A2TIK8	Integrin beta	1.1
S4	4.58	tr∣M4T4G3	Ryanodine receptor	1.3	R1 and R3	6.62	tr∣A0A1Q1MKI0	Prophenoloxidase PPO1b	1.7
S3	7.72	tr∣Q06GJ0	Beta-hexosaminidase	2.7	R2	5.87	tr∣A0A1B2AQF4	Trehalase	1.1
S2	2.88	tr∣M4Q143	NADH-ubiquinone oxidoreductase	3.1					
S2	21.42	tr∣Q7K403	Acyl-CoA delta-9 desaturase	5.1					
S3 and S4	1.84	tr∣G1JT78	Alkaline phosphatase	1.3					

Table S2. List of common Cry1Ah binding protein of BBMVs from susceptible and resistant strains of *Ostrinia furnacalis*.

ACB-BtS Midgut Fractions					ACB-AhR Midgut Fractions				
**Fraction**	**Score**	**Accession Number**	**Protein Description**	**Sequence Coverage %**	**Fraction**	**Score**	**Accession Number**	**Protein Description**	**Sequence Coverage %**
S1	65.3	tr∣B5A8X9	Aminopeptidase	7.2	R1	22.56	tr∣A0A0A7BYG4	Elongation factor 1 alpha	9.6
	20.09	tr∣D2Y440	Actin	19.9		19.68	tr∣E5LEV4	Heat shock 70	4.7
	17.09	tr∣Q8WB29	Cytochrome c oxidase subunit 3	2.7		15	tr∣A0A0F7QEA3	Actin	20.4
	16.87	tr∣A0A0A7BYG4	Elongation factor 1 alpha (Fragment)	7.6		21.4	tr∣B5A8X9	Aminopeptidase	1.2
	15.68	tr∣E5LEV4	Heat shock 70 kDa	3		6.05	tr∣A0A1B4ZBI6	Odorant binding protein	5.9
	14.34	tr∣B5A8Y0	Aminopeptidase	4.1		3.26	tr∣Q2PQR1	Ubiquitin	10.6
	12.45	tr∣Q8W7D1	ATP synthase subunit a	3.4		2.9	tr∣A0A0A7DWF3	Arginine kinase	2
	6.48	tr∣E3W6T9	V-ATPase subunit A	3.9		2.44	tr∣A0A0E4B3V9	Odorant receptor	4.2
	5.28	tr∣A0A0A7BYG7	Glyceraldehyde-3-phosphate dehydrogenase	8.6		59.08	tr∣A7LIA2	Cadherin-like protein	3.7
	5.1	tr∣A0A0F7QEE5	Carboxylic ester hydrolase	1.3		11.56	tr∣A0A0B6VK98	Uncharacterized protein	2
	4.66	tr∣Q8WB23	Cytochrome	2.4		6.99	tr∣A0A0F7QIG2	Ionotropic receptor	1.1
	3.97	tr∣A0A0F7QIG2	Ionotropic receptor	2.4		13.74	tr∣A0A0A7BYG4	Elongation factor 1 alpha	14.7
	3.84	tr∣Q2PQR1	Ubiquitin	24.3		12.4	tr∣Q8W7D1	synthase subunit a	3.1

	3.69	tr|A0A1B4ZBI6	Odorant binding protein	6		10.17	tr|Q8WB24	NADH dehydrogenase subunit 6	5.6
	2.95	tr|A0A0A7BYS7	Glutathione-S transferase (Fragment)	4.3		9.14	tr|Q8WB23	Cytochrome b	9.4
	2.9	tr|A0A0A7DWF3	Arginine kinase	2		8.85	tr|Q2V6H3	Chitin synthase	0.5
	2.34	tr|A0A0A7BYM5	Glutathione-S transferase	6		8.45	tr|A0A0F7QJX1	Carboxylic ester hydrolase	2
	2.13	tr|E0XJK4	Heat shock protein 90	1					
	7.59	tr|A0A1Q1MKI5	Prophenoloxidase PPO1a	3.1	R2	64.86	tr|B5A8X9	Aminopeptidase	15
	1.65	tr|A0A0B6VK98	Uncharacterized protein	2		45.15	tr|A0A0S2C6J0	Actin	14.1
	1.44	tr|B4YIR0	Chitin synthase	2.2		59.08	tr|A7LIA2	Cadherin-like protein	3.2
						28.02	tr|A0A0A7BYG4	Elongation factor 1 alpha	7.1
S2	240.08	tr|D0UYB1	Aminopeptidase	44.2		6.46	tr|B5A8X9	Aminopeptidase	1.5
	181.29	tr|A7YAH7	Aminopeptidase	27.4		17.85	tr|A0A0A7BYG7	Glyceraldehyde-3-phosphate dehydrogenase	4.5
	123.85	tr|B5A8Y0	Aminopeptidase	19.9		14.97	tr|A7YAH7	Aminopeptidase	2.3
	49.61	tr|A7LIA2	Cadherin-like protein	7.6		13.21	tr|E5LEV4	Heat shock 70 kDa	1.7
	29.97	tr|E3W6T9	V-ATPase subunit A	17.7		11.56	tr|A0A0B6VK98	Uncharacterized protein	2
	18.31	tr|A5JJU1	Aminopeptidase	23.9		7.31	tr|A0A0A7DWF3	Arginine kinase	2
	14.21	tr|E5LEV4	Heat shock 70 kDa	2.8		6.72	tr|Q2PQR1	Ubiquitin	11.8
	14.18	tr|A0A0S2C6J0	Actin	18.5		5.95	tr|L7QRW6	Period	2.2
	6.24	tr|Q8WB29	Cytochrome c oxidase subunit	5.3		5.77	tr|E3W6T9	V-ATPase subunit A	1.5
	5.9	tr|D0UYB2	Aminopeptidase	40.2		5.73	tr|A0A0F7QEC7	Aldehyde oxidase	1.4
	5.18	tr|A0A0F7QEE5	Carboxylic ester hydrolase	1.3		5.71	tr|A0A1Q1MKI5	Prophenoloxidase PPO1a	3.6
	1.7	tr|Q2PQR1	Ubiquitin	28.9		5.68	tr|A0A0E4B3V9	Odorant receptor	2
	1.68	tr|A0A0A7BYG7	Glyceraldehyde-3-phosphate dehydrogenase	2.1					
	1.6	tr|A0A1B4ZBI6	Odorant binding protein 9	5.9	R3	244.29	tr|B5A8X9	Aminopeptidase	28.9
	1.3	tr|M1RM07	Elongation factor-1 alpha	14.1		66.82	tr|A7YAH7	Aminopeptidase	9.2
	1.04	tr|Q8WB31	Cytochrome c oxidase subunit 1	1.6		65.03	tr|E3W6T9	V-ATPase subunit A	14.3

	0.95	tr∣A0A0E4B5I4	Odorant receptor	1.7		54.05	tr∣B5A8Y0	Aminopeptidase	10.6
	0.57	tr∣A0A0A7BYS7	Glutathione-S transferase	3.2		27.18	tr∣D2Y440	Actin	30.6
	0.49	tr∣L7QRW6	Period	2.2		25.37	tr∣D0UYB2	Aminopeptidase	7.7
	0.49	tr∣A0A0E3VLQ5	Odorant receptor	2.3		21.4	tr∣B2LS41	Aminopeptidase	26.9
	0.45	tr∣M1RTC5	Elongation factor-1 alpha	16.7		21.13	tr∣A0A0S2C6J0	Actin	23.9
						14.45	tr∣A0A0A7BYG4	Elongation factor 1 alpha	7.1
S3	323.31	tr∣E3W6T9	V-ATPase subunit A	71.5		13.98	tr∣E5LEV4	Heat shock 70 kDa	2.8
	95.44	tr∣B5A8X9	Aminopeptidase	21.7		13.91	tr∣A0A0A0YWU7	Arginine kinase	5.6
	50.77	tr∣E5LEV4	Heat shock 70 kDa	28.2		6.29	tr∣A0A0F7QIG2	Ionotropic receptor	1.1
	37.45	tr∣A7YAH7	Aminopeptidase	12.2		6.09	tr∣Q8WB29	Cytochrome c oxidase subunit 3	2.7
	30.53	tr∣E0XJK4	Heat shock protein 90	17.3		5.71	tr∣J7FBQ4	Odorant receptor	2.4
	17.39	tr∣B5A8Y0	Aminopeptidase	8		5.6	tr∣M1RTC5	Elongation factor-1 alpha	5.6
	14.82	tr∣A0A0A7BYG4	Elongation factor 1 alpha	14.7		5.6	tr∣A0A0F7QEJ4	Sensory neuron membrane protein 1	1.5
	9.53	tr∣A0A1L7B974	Serpin 5	12.2		2.43	tr∣A0A1L7B973	Serine proteinase inhibitor 2	17.5
	12.66	tr∣A0A0F7QJX1	Carboxylic ester hydrolase	9.6		14.59	tr∣A0A1L7B974	Serpin 5	12.4
	9.12	tr∣B2LS41	Aminopeptidase	21.7					
	7.98	tr∣D0UYB2	Aminopeptidase	5.9	R4	231.15	tr∣E3W6T9	V-ATPase subunit A	27.1
	7.98	tr∣D2Y440	Actin (Fragment)	19.1		164.37	tr∣B2LS41	Aminopeptidase	16.6
	7.83	tr∣A0A0A7DWF3	Arginine kinase	8.5		48.18	tr∣D2Y440	Actin	30.2
	4.99	tr∣M1RTC5	Elongation factor-1 alpha	22.3		46.51	tr∣B5A8X9	Aminopeptidase	16.8
	4.83	tr∣A0A0S2C6J0	Actin	14.1		43.97	tr∣A0A0S2C6J0	Actin	28.3
	3.62	tr∣M1RM07	Elongation factor-1 alpha	19.4		37.32	tr∣E0XJK4	Heat shock protein 90	6.3
	2.96	tr∣Q2PQR1	Ubiquitin	24.9		30.01	tr∣E5LEV4	Heat shock 70 kDa	6.3
	2.64	tr∣Q8WB31	Cytochrome c oxidase subunit 1	1.6		24.14	tr∣A0A0A7BYG4	Elongation factor 1 alpha	7.1
						22.07	tr∣F6MEP1	Storage protein	6.1
S4	258.25	tr∣E3W6T9	V-ATPase subunit A	49.2		8.94	tr∣Q8WB29	Cytochrome c oxidase subunit 3	2.7
	49.45	tr∣B5A8X9	Aminopeptidase	14.4		8.02	tr∣M1RTC5	Elongation factor-1 alpha	9.4
	24.74	tr∣A7YAH7	Aminopeptidase	8.7		7.72	tr∣A0A0F7QEE5	Carboxylic ester hydrolase	1.3
	23.7	tr∣A0A1L7B973	Serine proteinase inhibitor 2	14.6		6.74	tr∣A0A1B4ZBI6	Odorant binding protein 9	5.9

<table>
<tr><td>19.1</td><td>tr|A0A0F7QJX1</td><td>Carboxylic ester hydrolase</td><td>13.3</td><td>6.65</td><td>tr|A0A0A7BYG7</td><td>Glyceraldehyde-3-phosphate dehydrogenase</td><td>2.1</td></tr>
<tr><td>17.18</td><td>tr|A0A0F7QIF1</td><td>Carboxylic ester hydrolase</td><td>15</td><td>6.4</td><td>tr|A0A1Q1MKI5</td><td>Prophenoloxidase PPO1a</td><td>1.2</td></tr>
<tr><td>10.56</td><td>tr|E0XJK4</td><td>Heat shock protein 90</td><td>7.8</td><td>5.98</td><td>tr|A0A0A7BYS7</td><td>Glutathione-S transferase</td><td>3.2</td></tr>
<tr><td>6.84</td><td>tr|A0A0S2C6J0</td><td>Actin</td><td>17.9</td><td></td><td></td><td></td><td></td></tr>
<tr><td>6.06</td><td>tr|A0A0B6VQ49</td><td>Uncharacterized protein</td><td>42.7</td><td></td><td></td><td></td><td></td></tr>
<tr><td>5.24</td><td>tr|D2Y440</td><td>Actin</td><td>19.1</td><td></td><td></td><td></td><td></td></tr>
<tr><td>4.9</td><td>tr|A0A0A7BYG4</td><td>Elongation factor 1 alpha</td><td>11.2</td><td></td><td></td><td></td><td></td></tr>
<tr><td>4.37</td><td>tr|A0A0A7DWF3</td><td>Arginine kinase</td><td>8.5</td><td></td><td></td><td></td><td></td></tr>
<tr><td>4.23</td><td>tr|B5A8Y0</td><td>Aminopeptidase</td><td>2.2</td><td></td><td></td><td></td><td></td></tr>
<tr><td>3</td><td>tr|M1RM07</td><td>Elongation factor-1 alpha</td><td>14.1</td><td></td><td></td><td></td><td></td></tr>
<tr><td>1.81</td><td>tr|Q8WB29</td><td>Cytochrome c oxidase subunit 3</td><td>2.7</td><td></td><td></td><td></td><td></td></tr>
<tr><td>1.67</td><td>tr|E5LEV4</td><td>Heat shock 70 kDa</td><td>1.1</td><td></td><td></td><td></td><td></td></tr>
<tr><td>1.48</td><td>tr|Q2V6H3</td><td>Chitin synthase</td><td>3</td><td></td><td></td><td></td><td></td></tr>
<tr><td>1.33</td><td>tr|D2KWQ3</td><td>Odorant receptor</td><td>2.5</td><td></td><td></td><td></td><td></td></tr>
<tr><td>1.08</td><td>tr|B2LS41</td><td>Aminopeptidase</td><td>12.7</td><td></td><td></td><td></td><td></td></tr>
<tr><td>0.89</td><td>tr|A0A1L7B974</td><td>Serpin 5</td><td>22.9</td><td></td><td></td><td></td><td></td></tr>
<tr><td>0.87</td><td>tr|A0A1U8ZSW0</td><td>Storage protein</td><td>1.2</td><td></td><td></td><td></td><td></td></tr>
</table>
